# Effectiveness of Direct Laser Interference Patterning and Peptide Immobilization on Endothelial Cell Migration for Cardio-Vascular Applications: An In Vitro Study

**DOI:** 10.3390/nano12071217

**Published:** 2022-04-05

**Authors:** Romain Schieber, Carlos Mas-Moruno, Federico Lasserre, Joan Josep Roa, Maria-Pau Ginebra, Frank Mücklich, Marta Pegueroles

**Affiliations:** 1 Biomaterials, Biomechanics and Tissue Engineering Group, Department of Materials Science and Engineering, Barcelona East School of Engineering (EEBE), Universitat Politècnica de Catalunya (UPC), Av. Eduard Maristany, 10-14, 08019 Barcelona, Spain; romain.schieber@upc.edu (R.S.); carles.mas.moruno@upc.edu (C.M.-M.); maria.pau.ginebra@upc.edu (M.-P.G.); 2 Barcelona Research Center in Multiscale Science and Engineering, Universitat Politècnica de Catalunya (UPC), 08019 Barcelona, Spain; joan.josep.roa@upc.edu; 3 Chair of Functional Materials, Faculty of Natural Sciences and Technology, Saarland University, 66123 Saarbrücken, Germany; lasserre@matsci.uni-sb.de (F.L.); muecke@matsci.uni-sb.de (F.M.); 4 Structural Integrity, Micromechanics and Reliability of Materials Group, Department of Materials Science and Metallurgical Engineering, Barcelona East School of Engineering (EEBE), Universitat Politècnica de Catalunya (UPC), 08019 Barcelona, Spain; 5 Institute for Bioengineering of Catalonia (IBEC), 08028 Barcelona, Spain

**Keywords:** direct laser interference patterning (DLIP), cobalt-chromium alloy, biofunctionalization, cell adhesive peptides, endothelial cell migration

## Abstract

Endothelial coverage of an exposed cardiovascular stent surface leads to the occurrence of restenosis and late-stent thrombosis several months after implantation. To overcome this difficulty, modification of stent surfaces with topographical or biochemical features may be performed to increase endothelial cells’ (ECs) adhesion and/or migration. This work combines both strategies on cobalt-chromium (CoCr) alloy and studies the potential synergistic effect of linear patterned surfaces that are obtained by direct laser interference patterning (DLIP), coupled with the use of Arg-Gly-Asp (RGD) and Tyr-Ile-Gly-Ser-Arg (YIGSR) peptides. An extensive characterization of the modified surfaces was performed by using AFM, XPS, surface charge, electrochemical analysis and fluorescent methods. The biological response was studied in terms of EC adhesion, migration and proliferation assays. CoCr surfaces were successfully patterned with a periodicity of 10 µm and two different depths, D (≈79 and 762 nm). RGD and YIGSR were immobilized on the surfaces by CPTES silanization. Early EC adhesion was increased on the peptide-functionalized surfaces, especially for YIGSR compared to RGD. High-depth patterns generated 80% of ECs’ alignment within the topographical lines and enhanced EC migration. It is noteworthy that the combined use of the two strategies synergistically accelerated the ECs’ migration and proliferation, proving the potential of this strategy to enhance stent endothelialization.

## 1. Introduction

Cardiovascular stents are devoted to solve coronary artery disease (CAD), which is characterized by the narrowing of the artery, due to fatty and lipid deposits beneath endothelium. The first-generation stents, bare metal stents (BMSs), were fabricated with inert materials, such as stainless steel (316L), cobalt-chromium (Co-Cr), platinum-iridium (Pt-Ir) alloys, tantalum (Ta) or nitinol (Ni-Ti), with the final goal of maintaining the artery open [1,2]. Conversely, the second generation of stents, drug eluting stents (DESs), incorporated a polymer coating, which delivers drugs to reduce intima hyperplasia [2,3]. For example, the Supralimus stent (Sahajanand Medical Technologies Ltd., Surat, India) has shown excellent results by reducing in-stent restenosis thanks to the action of sirolimus, which primarily inhibits smooth muscle cells’ (SMCs) proliferation [4,5]. However, current DESs delay endothelialization, due to the delivery of non-specific anti-proliferative or anti-inflammatory drugs [5]. Moreover, in some cases, an increase of late stent thrombosis has been detected, as well, mainly dependent on the strut thickness and the polymer coating’s durability [6,7].

In general, bioactive surface treatments allow biomaterials to ameliorate their integration into the body and accelerate the healing of damaged tissues [8,9]. Thus, a feasible approach to enhance endothelial cells’ (ECs) adhesion and migration is to biofunctionalize the implant surface. This strategy commonly consists of the immobilization of specific biomolecules onto the inert biomaterial to create a cell instructive microenvironment that mimics the extracellular matrix (ECM) [10,11,12]. The way cells adhere to the implant surface and exchange biochemical signals is through cell membrane receptors. ECs express integrins, which have the capacity to recognize and bind to ECM proteins of ECs (e.g., collagen 1, laminin, heparin and fibrin) [13]. Biofunctionalization of the stent surface with biomolecules derived from ECM components, therefore, represents an attractive strategy to promote integrin-mediated binding of ECs and improve re-endothelialization. Early studies investigated the use of full-length ECM proteins to coat implant surfaces, such as collagen, heparin [14,15,16], laminin [17] or fibrin [18]. Although this approach was proven to be successful to enhance cell adhesion on the functionalized surfaces, the use of proteins is limited due to several disadvantages, such as their poor enzymatic stability, immunogenicity and conformation-dependent efficiency [19,20]. Most of these drawbacks may be overcome by using short ECM-derived peptides that contain the cell adhesive motifs present in the natural proteins. Indeed, short synthetic peptides are not immunogenic, have lower steric requirements and their use is exempt from infection risks [21]. As an example, the well-known Arg-Gly-Asp sequence (RGD) is present in fibronectin and other ECM proteins and is critical for mediating cell adhesion [19,22]. The RGD motif has been extensively used to enhance ECs adhesion onto biomaterials [10,23,24]. However, RGD is not specific for ECs, and it may also influence the adhesion of SMCs and platelets. Thus, other ECM-derived peptide sequences have been isolated and grafted onto biomaterials to specifically enhance ECs’ related processes, e.g., the Arg-Glu-Asp-Val (REDV) peptide [25,26,27], which is a fibronectin-derived recognition sequence, specifically targeting ECs α_4_β_1_ integrin [28], or Tyr-Ile-Gly-Ser-Arg (YIGSR), a motif that is present in laminin and promotes ECs’ adhesion [25,27,29]. In particular, the laminin-derived YIGSR peptide has demonstrated improved EC attachment, spreading and resistance to shear stress from blood flow [29,30,31]. Moreover, we previously reported [32] that the immobilization of RGD, REDV and YIGSR peptide sequences onto cobalt-chromium (CoCr) alloy surfaces enhanced ECs’ adhesion and proliferation, without a significant increase in SMCs adhesion. 

At the artery, endothelialization is mainly achieved through ECs’ migration from the healthy endothelium situated at both extremities of the stent. Different approaches have tried to modify the stent topography with a linear patterning in order to mimic the orientation of ECs at the coronary artery lumen [33,34,35,36,37]. A large number of techniques have been applied to modify the surface topography of cardiovascular implants, including metallographic grinding, plasma etching, photolithographic etching and UV lithography, among others [38,39,40,41]. However, most of the mentioned processing techniques involve multiple steps of preparation, and they sometimes include coatings steps. On the contrary, direct laser interference patterning (DLIP) requires only one single processing step, and it can be applied to a wide range of materials [42]. In this regard, we recently demonstrated that 600 nm–deep linear patterns obtained by DLIP on CoCr alloy surfaces induced the alignment and migration of ECs [33]. 

Based on these precedents, the combination of topographical and chemical modifications holds promise to maximize and possibly synergize positive cell responses at the biomaterial surface [8]. Indeed, such a strategy has been recently applied to dental zirconia, proving useful to tune mesenchymal stem cell adhesion and osteogenic differentiation [43]; however, to the best of our knowledge, this approach has never been investigated for ECs on stent surfaces. Within this context, the aim of this work was to modify CoCr alloy surfaces with a combination of linear patterning obtained by DLIP and biofunctionalization with RGD and YIGSR peptides in order to study the effect of both treatments in ECs’ adhesion, migration and proliferation. Linear-patterned CoCr surfaces were characterized topographically by atomic force microscopy (AFM), and electrochemical assays were applied to determine their corrosion-resistance properties. RGD and YIGSR peptides were covalently immobilized by silanization, and the obtained coating was characterized by Fourier-transform infrared spectroscopy (FTIR), X-ray photoelectron microscopy (XPS), AFM and fluorescence microscopy. Finally, the effect of surface modifications on the biological response was evaluated by studying the ECs’ adhesion, migration and proliferation on the patterned and biofunctionalized surfaces.

## 2. Materials and Methods

### 2.1. CoCr Alloy Surfaces

CoCr disks of 10 mm in diameter and 2 mm in thickness were obtained by turning from a bar of CoCr alloy ASTM F90 (51% Co, 20% Cr, 15% W,10% Ni and 3% Fe) (Technalloy, Sant Cugat del Vallès, Spain), grinded and mirror-like polished. Disks were ultrasonically cleaned with cyclohexane, isopropanol, ethanol, Milli-Q water and acetone for 5 min and dried with nitrogen for storage.

### 2.2. DLIP Patterning

Patterned CoCr surfaces were obtained by applying a Nd:YAG laser (Spectra Physics Quanta-Ray PRO 290, Spectra-Physics, Milpitas, CA, USA) with a fundamental wavelength (λ) of 1064 nm. Samples were structured at a wavelength λ of 355 nm (third harmonic generation) with a repetition rate of 10 Hz and pulse duration of 10 ns. The result was a line-like interference pattern with a periodicity (P) of 10 µm related to the angle 2α, between the two laser beams, through the following equation: P = λ/2 ∗ sin(α) [42]. Laser fluency was varied to generate different pattern depths (D). A 0.81 J·cm^−2^ fluence was used to obtain a low-depth pattern (coded as 10L with D < 100 nm), and 2.42 J∙cm^−2^ fluence was used to obtain a high-depth pattern (coded as 10H with D > 700 nm). Further details on the experimental setup and on the properties of the obtained patterns have already been published elsewhere [33,42]. Polished and non-patterned CoCr surfaces were labelled as FLAT.

### 2.3. Functionalization of CoCr Surfaces

#### 2.3.1. Solid-Phase Peptide Synthesis

**Figure 1:**
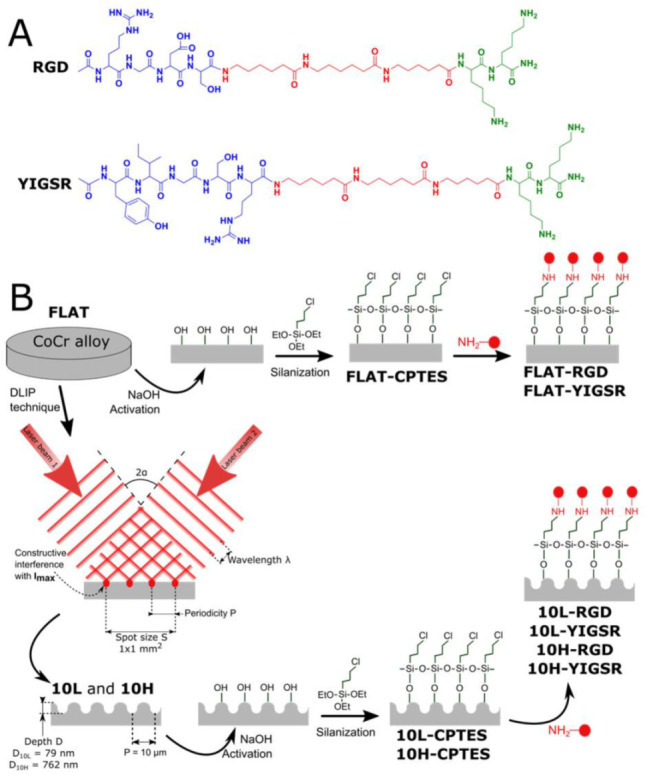
(**A**) Chemical structure of RGD and YIGSR peptides. Green: anchoring sequence (Lys-Lys); Red: spacer units (Ahx-Ahx-Ahx). Blue: functional sequences (Arg-Gly-Asp-Ser and Tyr-Ile-Gly-Ser-Arg). (**B**) Scheme of the DLIP and biofunctionalization steps to obtain the different patterned and/or biofunctionalized CoCr surfaces (FLAT—polished CoCr; 10L—low-depth linear-patterned CoCr surfaces; 10H—high-depth linear-patterned CoCr surfaces; FLAT–CPTES, 10L–CPTES and 10H–CPTES—FLAT, 10L and 10H, followed by CPTES silanization; FLAT–RGD, 10L–RGD and 10H–RGD—FLAT, 10L and 10H, followed by CPTES silanization and biofunctionalization with RGD; FLAT–YIGSR, 10L–YIGSR and 10H–YIGSR—FLAT, 10L and 10H, followed by CPTES silanization and biofunctionalization with YIGSR).

The linear peptides RGDS and YIGSR were synthesized in solid phase, following the Fmoc/tBu strategy, using Fmoc-Rink amide 4-methylbenzhydrylamine (MBHA resin) as solid support (300 mg, loading of 1.0 mmol/g), according to previously reported methods [44]. The two functional sequences were synthesized containing three 6 aminohexanoic acid (Ahx) units as spacer system and two lysine (Lys) residues, which served as anchors (Figure 1A). Moreover, the two peptides were synthesized with an additional 5(6)-carboxyfluorescein (CF) at their N-termini for fluorescence microscopy. The peptides were purified by semi-preparative HPLC (Waters, Milford, MA, USA) (XBridge C18 column, 19 mm × 150 mm, 5 μm) and characterized by analytical HPLC (Waters) (XBridge C18 column, 4.6 mm × 100 mm, 3.5 μm) and MALDI–TOF Voyager DE RP spectrometer (Applied Biosystems, Foster City, CA, USA) (Table 1). All chemicals required for the synthesis were obtained from Iris Biotech GmbH (Marktredwitz, Germany) and Sigma-Aldrich (Burlington, MA, USA).

#### 2.3.2. Protocol of Immobilization

The peptides were covalently immobilized on the CoCr surfaces through a three-step strategy consisting of activation, silanization and peptide immobilization (Figure 1B).

First, surfaces were activated with 5 M NaOH solution for 2 h, at room temperature (RT), and cleaned twice in distilled water for 30 min, under ultrasonication. Afterward, activated samples were silanized in 10 mL solution of 0.5 M 3 chloropropyltriethoxysilane (CPTES) (Sigma-Aldrich, Burlington, MA, USA) and 0.05 M N,N diisopropylethylamine (DIEA) in anhydrous toluene at 90 °C for 1 h, under nitrogen atmosphere and vigorous stirring. Silanized samples were ultrasonically rinsed in cyclohexane, isopropanol, Milli-Q distilled water and acetone for 15 min each, and finally dried with nitrogen (samples coded as FLAT–CPTES, 10L–CPTES and 10H–CPTES). Finally, silanized samples were immersed overnight in 100 µM peptide solutions (samples coded as FLAT–RGD, 10L–RGD, 10H–RGD, FLAT–YIGSR, 10L–YIGSR and 10H–YIGSR) in phosphate-buffered saline (PBS), with a pH 10.0 at RT. The use of this basic pH is necessary to deprotonate the amino groups of the lysine residues, ensuring a nucleophilic attack to the C-Cl groups of CPTES, and to neutralize the HCl generated during the process. This basic pH does not affect the integrity of the peptide backbone [45,46,47]. The following day, the samples were washed three times with distilled water.

### 2.4. Physicochemical Characterization of the Patterned Surfaces

Surface morphology and roughness of CoCr samples were analyzed by atomic force microscopy (AFM) (Dimension 3100, Veeco Digital Instruments, Plainview, NY, USA). Topographic, error signal mode and phases images were recorded simultaneously in tapping-mode, in air, using a silicon Tap150al-G cantilever (NanoWorld Group, Neuchâtel, Switzerland) at a scan rate of 1 Hz. Five areas of 50 × 50 µm^2^ were analyzed for FLAT, 10L and 10H CoCr surfaces. Obtained images were studied by using WSxM software [48]. The topographical profile, as well as the root-mean-square roughness (Rq), were obtained from topographic images: Rq-micro from areas of 50 × 50 µm^2^ and Rq-nano from areas of 1 × 1 µm^2^.

Electrochemical characterization was performed with a potentiostat PARSTAT 2273 (Princeton Applied Research, Oak Ridge, TN, USA) with a Faraday cage. Measurements were controlled with the software PowerSuite Electrochemical (Princeton Applied Research, Oak Ridge, TN, USA). A saturated calomel electrode (SCE, Koslow Scientific Company, Englewood, NJ, USA) was used as a reference electrode, and a Hank’s balanced salt solution (Sigma-Aldrich, Burlington, MA, USA) was used as electrolyte solution. Open-circuit-potential assays were performed by following the standard ISO 10993-15 [49] in order to determine the corrosion potential of patterned CoCr surfaces. Degradation products of CoCr samples were evaluated after immersion in the Hank’s balanced salt solution at 37 °C for seven days. Released ions to the medium were measured by mass spectroscopy ICP–MS (Agilent 7500CE, Agilent Technologies, Santa Clara, CA, USA), as indicated by the standard ISO 10993-15 [49].

A streaming potential instrument (Surpass Electrokinetic Analyzer, Anton Paar, Graz, Austria) with an adjustable gap cell was used to obtain the zeta-potential of FLAT, 10L and 10H surfaces. Measurements were performed with a 1 mM KCl solution as electrolyte and the pressure ramp run up to a maximum of 400 mbar. The initial electrolyte solution was adjusted to pH 9.0 using 0.1 M KOH solution and titrated by adding 0.1 M HCl down to pH 3.0. The isoelectric point (IEP) and the surface charge at pH 7.4 were obtained using the VisioLab software (Anton Paar, Graz, Austria).

### 2.5. Physicochemical Characterization of the Biofunctionalized Surfaces

Silanized and biofunctionalized CoCr surfaces were chemically characterized with attenuated total reflectance–Fourier-transform infrared (ATR–FTIR) spectroscopy, using a Nicolet 6700 FTIR spectrometer (Thermo Fisher Scientific, Waltham, MA, USA) combined with the ATR Smart Saga furnished with a diamond crystal. All samples’ spectra were analyzed with OMNIC 8 software (Thermo Fisher Scientific, Waltham, MA, USA). 

To characterize the silane and peptides layers, FLAT and biofunctionalized CoCr surfaces were analyzed by using AFM. AFM conditions were similar to those previously described in Section 2.4. Only the scanning velocity and area were changed to 0.5 Hz and 2 × 2 µm^2^, respectively. The topographical profile and roughness Rq-nano were obtained from height images. Phase images were used to extract the phases distribution and calculate the percentage of area covered by non-metallic substances.

X-ray photoelectron spectroscopy (XPS): Measurements were performed with a SPECS Surface Nano Analysis GmbH (Berlin, Germany) equipped with a Mg anode X50 source operating at 150 W and a Phoibos 150 MCD-9 detector. The elements present on the surface were evaluated by low-resolution survey spectra, whereas high-resolution of some elements (C 1s, O 1s, Cr 2p, Co 2p, N 1s and Cl 2p) were measured with a pass energy of 25 eV at 0.1 eV steps, at a pressure below 7.5 × 10^−9^ mbar. All binding energies were referred to the C1s signal at 284.8 eV. Casa XPS software (Version 2.3.16, Casa Software Ltd., Teignmouth, UK) was used to do fitting and peak integration of spectra.

The stability of the peptide layer toward sterilization and ultrasonication treatments was analyzed by fluorescence. To this end, samples were functionalized with fluorescent peptides RGD–CF and YIGSR–CF and subsequently subjected to either sterilization by gamma radiation (AragoGamma, La Roca del Vallès, Spain) using a 25 kGy dose (samples FLAT–RGD–RG and FLAT–YIGSR–RG) or ultrasonication during 1h in PBS (samples FLAT–RGD–US and FLAT–YIGSR–US). Then, the fluorescent immobilized peptides before and after stability treatments were extracted with 500 µL of 0.2 M NaOH for 2 h, at RT [50]. The fluorescence of the extracted solution was measured with a spectrofluorophotometer (Infinite M200 PRO NanoQuant Microplate reader, Tecan, Männedorf, Switzerland) at 492 nm excitation and 517 nm emission wavelengths. The quantity of attached peptides was determined by comparison with a standard curve.

### 2.6. Biological Characterization

Biological characterization is dedicated to compare the effect of topography and functionalization on polished and non-functionalized CoCr surfaces. It has been previously demonstrated that CPTES alones does not promote cell adhesive events [51], and, thus, this control was not included in the present biological studies.

#### 2.6.1. Endothelial Cells

Cellular experiments were conducted by using human umbilical vein endothelial cells (HUVECs) (Lonza Group Ltd., Basel, Switzerland) grown in EC basal medium (EBM^®^) supplemented with 4% (*v*/*v*) fetal bovine serum (FBS), 0.1% (*v*/*v*) gentamicin sulfate amphotericin (GA-1000), 0.4% (*v*/*v*) recombinant human fibroblast growth factor (rhFGF), 0.1% (*v*/*v*) recombinant human epidermal growth factor (rhEGF), 0.1% (*v*/*v*) ascorbic acid, 0.1% (*v*/*v*) vascular endothelial growth factor (VEGF), 0.1% (*v*/*v*) recombinant Long R insulin (R3-IGF-1) and 0.04% (*v*/*v*) hydrocortisone (EBM and all supplements were obtained from Lonza, Basel, Switzerland). Cells were maintained at 37 °C in a humidified atmosphere containing 5% (*v*/*v*) CO_2_, changing culture medium thrice a week. All experiments were conducted using HUVECs at passages five to seven. All cellular studies were performed by using triplicates and repeated at least in two independent assays to ensure reproducibility. 

#### 2.6.2. Cell Adhesion

Previous to cell adhesion assay, CoCr samples were sterilized in ethanol 70% for 20 min at RT. Next, samples were placed in 48-well plates, and 30,000 cells/disk were seeded and allowed to attach in serum-free medium. Samples were incubated overnight at 37 °C, rinsed thrice with PBS to remove non-adherent cells and, nuclei and actin fibers were stained by immunofluorescence. Briefly, adherent cells were fixed for 30 min with 4% (*w*/*v*) paraformaldehyde (PFA). Then cells were permeabilized with 500 μL/disk of 0.05% (*w*/*v*) triton X-100 in PBS for 20 min and blocked with 1% BSA (*w*/*v*) in PBS for 30 min. Between every step, samples were washed three times with PBS-Gly (PBS containing 20 mM of glycine) for 5 min each. Next, cells were incubated in the dark with phalloidin-rhodamine (1:300; Invitrogen, Thermo Fisher Scientific, Waltham, MA, USA) for one hour. Nuclei of cells were also stained with 500 μL/disk of DAPI (1:1000) in PBS-Gly for 2 min in the dark. Finally, samples were examined by fluorescence microscopy (AF7000, Leica Microsystems, Wetzlar, Germany). Cell number and morphology parameters were studied by using FIJI software [52], obtaining the cell number, cell mean area and percentage of aligned cells for each surface. Cells were considered aligned if the angle between the long axis and the pattern was <30°. Once analyzed, CoCr samples were ultrasonically cleaned in PBS, and adhered cells were fixed with glutaraldehyde (G400-4, Sigma-Aldrich, Burlington, MA, USA) and dehydrated through immersion in different solutions of ethanol in order to observe, in more detail, the cells’ shape by field emission scanning electron microscopy (FE-SEM) with a Zeiss Neon40 FE-SEM microscope (Carl Zeiss NTS GmbH, Oberkochen, Germany). For each sample, five images were taken at a working distance of 6 mm and a potential of 5 kV. 

#### 2.6.3. Wound Healing Migration Study

To evaluate the process of wound healing and cell migration on patterned and functionalized CoCr surfaces, HUVECs were stained with the cell membrane fluorescent linker PKH67 (PKH67-GL, Sigma-Aldrich, Burlington, MA, USA) 24 h prior to wound-healing assays. The wound-healing processes were reproduced by placing an Ibidi culture-insert (Ibidi, Gräfelfing, Germany) on top of each sample. On the patterned surfaces, the Ibidi insert was placed, keeping the gap perpendicular to the pattern’s lines. Thus, a suspension of 20,000 labeled cells was placed at both chambers of the insert, with complete medium, and incubated for 24 h until reaching a confluent HUVEC adhesion. After, the insert was removed, creating a cell-free gap of 500 ± 50 µm. The surfaces were cleaned twice with PBS to remove non-adhered cells and then filled with serum-free medium. HUVEC cell migration was recorded by using a fluorescence stereomicroscope (Leica MZ16F, Leica microsystems, Wetzlar, Germany) combined with the camera DFC 300 FX (Leica Microsystems, Wetzlar, Germany). Gap images were acquired for all the samples at 0 and 24 h. The non-covered area for each CoCr disk was calculated with FIJI software, and the percentage of recovery was calculated for each series.

#### 2.6.4. Cells Proliferation

To study the proliferation of cells on the substrates, 10,000 HUVECs/well were plated on samples in serum-free medium and incubated as previously described for cell adhesion assays. Four hours post-seeding, medium was aspired and replaced with complete medium. After 2, 5 and 8 days of incubation, cell number was evaluated with the Alamar Blue assay (Invitrogen Life Technologies, Merelbeke, Belgium). Briefly, Alamar Blue–containing medium (10% (*v*/*v*)) was added for 2 h, and fluorescence of the dye was quantified according to the manufacturer instructions with a multimode microplate reader (Infinite M200 PRO, Tecan GroupLtd., Männedorf, Switzerland).

### 2.7. Statistical Analysis

The experimental data presented in this study are reported as mean value ± standard deviations (SDs). All physicochemical characterization and cell culture experiments were performed with n = 3 samples for each specimen group for each assay used in this study. A normality test (Shapiro–Wilk test) was performed to determine if the dataset was modeled by a normal distribution. Statistical significance between means was analyzed by an equality of variances test (ANOVA) test, followed by post hoc pair-wise comparison, using Tukey’s test, at *p* ≤ 0.05 (Minitab 16.2.2 Statistical Software, Minitab Inc., State College, PA, USA). 

## 3. Results and Discussion

Restenosis and late-stent thrombosis remain the most challenging limitations after stent placement. Different studies have proven the value of functional and intact endothelium in the prevention of restenosis and thrombosis [27]. In that sense, surface topographical modification attempts to alter the surface properties and provide the surface with specific functions, while not disturbing or damaging the bulk material properties. Previous works have developed surface topographical features by chemical vapor deposition (CVD) [53], anodization [54], electron-beam lithography [55], plasma-based dry etching [40] or femtosecond laser [56]. Moreover, in order to improve biocompatibility, inorganic coatings, polymeric coatings, organic–inorganic hybrid materials and biological coatings have been investigated as stent coatings [57,58]. In this work, we present a novel combination of linear patterning, obtained by DLIP, and biofunctional coating, with selected biomolecules, applied to the stent surface with the final goal to enhance surface endothelialization.

### 3.1. Fabrication of Linear Patterned CoCr by DLIP and Surface Characterization

**Figure 2:**
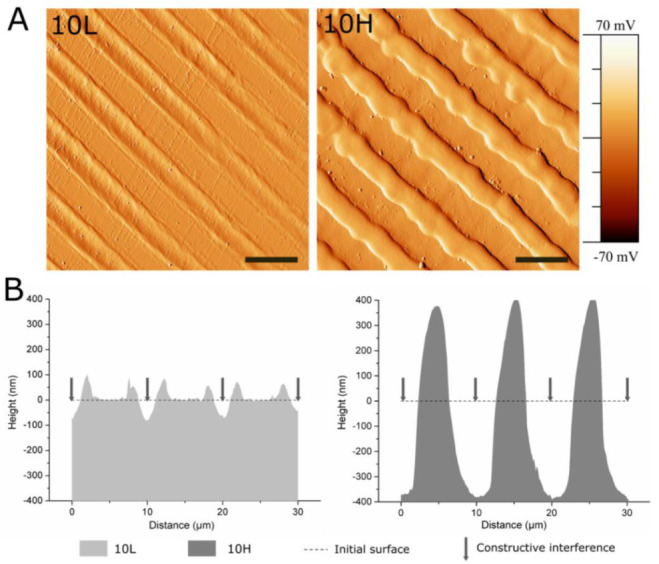
AFM analysis of 10L and 10H surfaces to characterize the patterns’ topographical properties. (**A**) AFM error signal mode images of 10L and 10H surfaces. Scale bar = 10 μm. (**B**) AFM topographical profiles of 10L and 10H surfaces in the direction perpendicular to the patterns lines. Dotted line indicates the estimated initial surface.

CoCr surfaces were topographically modified by using the DLIP technique. Linear patterns with a periodicity of 10 µm were obtained. Its size was slightly smaller than the ECs’ nucleus in order to induce ECs’ elongation and migration following the pattern direction. Figure 2 and Table 2 summarize the measured topographical parameters directly measured by AFM. By modifying the laser fluence, two different structures were obtained, low-depth patterned 10L (D ≈ 79 nm) and high-depth patterned 10H (D ≈ 762 nm) surfaces. As expected, the topographical modifications increased the micro-roughness, Rq-micro, of non-modified surfaces (FLAT) from 7.4 ± 1.3 nm up to 33.2 ± 3.7 nm for 10L surfaces and 303.6 ± 24.9 nm for 10H series. Moreover, the nano-roughness of FLAT samples and patterned surfaces, either at peaks or valleys, showed no statistical differences. AFM error signal mode images (Figure 2A) for the two different patterned surfaces confirmed that 10H surfaces presented homogenous grooves with clearly identifiable peaks and valleys; in contrast, on 10L surfaces, the low depths hindered the identification of peaks compared to valleys. Figure 2B presents the cross-section profiles of 10L and 10H surfaces directly extracted from the AFM topographic images, which allowed us to characterize the shape of the peaks. Indeed, 10H is characterized by a sinusoidal shape with valleys, where constructive interference occurs, and peaks, where destructive interference takes place and, thus, two melted material fronts were joined. On the contrary, 10L surfaces are composed of a low-depth valley where the constructive interferences met, creating two small peaks at both sides and a non-modified CoCr surface area, FLAT, where the beams interacted destructively. These two different configurations, called respectively single- and double-peak pattern surfaces, are usual for metallic surfaces modified by DLIP, as described in previous studies [33]. Such differences can be explained by the applied laser fluencies: high fluencies used for 10 H series, provided enough energy to melt a considerable amount of metal that flowed outside the constructive interference spots towards the destructive interference spots, due to the surface tension gradient resulting from the temperature gradient [59]. At the destructive interference spots, the advancing melt fronts join, creating the characteristic peaks. In the case of the 10L series, the applied low fluence melted a lower quantity of CoCr alloy, and the two fronts could not joint together, thus generating two small peaks surrounding the spots where the beams interfered constructively. In both surfaces, 10L and 10H, the high temperatures applied during DLIP transformed the crystalline oxide layer into a thicker amorphous oxide layer over the surface, as demonstrated by our previous study [33]. Thus, considering the low roughness of 10L surfaces, the 10L series could be considered mainly as surfaces with chemical changes and a negligible topographical pattern.

CoCr alloy ASTM F 90 is extensively used as stent material mainly due to its capacity of forming a naturally passive oxide layer that confers very good corrosion-resistance properties to the material. As surface patterning by DLIP could generate changes in the corrosion behavior of the materials, electrochemical assays and ion release tests were performed (Table 3). All studied series presented statistically equal corrosion potentials of approximately 1000 mV, suggesting that DLIP did not modify the corrosion resistance of the CoCr surfaces. Moreover, no corrosion pits were observed on the tested surfaces (not shown), thus validating DLIP as a treatment that does not modify the corrosion resistance observed in FLAT surfaces. Ion release assays were performed in Hank’s balanced salt solution at 37 °C during 7 days. The release of Ni ions, which are susceptible to allergy, was not significantly higher on 10L and 10H surfaces, compared to FLAT surface. The release of Co and Cr ions on 10L surfaces was slightly lower than on FLAT, indicating that 10L series are not toxic just as the CoCr alloys used in the current stents. On the other hand, the release of ions significantly increased on 10H series (Cr, 4.3 ± 0.3 ng; Co, 77.8 ± 8.6 ng) when compared to FLAT (Cr, 1.6 ± 0.3 ng; Co, 47.5 ± 16.0 ng). However, such a higher release should not be detrimental for the biological performance of these materials. Firstly, the high toxicity and carcinogenic properties of Cr are associated with its hexavalent form (chromium VI) [60], and we have previously shown that DLIP generated only Cr (III) at the CoCr alloy oxide layer [33]. Secondly, it should be taken into consideration that stents are placed in the artery and release metallic ions under blood-flow conditions. Thus, if we consider 5 liters as the normal blood quantity for an adult and the stent surface area being half of the area of the studied samples, the release of Cr and Co from 10H surfaces would be equivalent to 4.3 × 10^−4^ ng/mL and 7.8 × 10^−3^ ng/mL, respectively. These values are 100,000 and 15,000 times lower for Cr and Co, respectively, than the maximum concentrations recommended by the World Health Organization (Cr, 50 ng/mL; Co, 150 ng/mL) [61,62]. Consequently, even if the released ions were increased on 10H series compared to FLAT, the release of metallic ions after DLIP patterning is kept at non-toxic levels.

The zeta-potential studies indicated that the IEP of the FLAT and the patterned surfaces is around pH ~4.7. As the solution was more alkaline, the surfaces showed a steadily more negative zeta potential. The ζ at pH 7.4 is very similar on FLAT and 10L surfaces mainly because the roughness of 10L surfaces is negligible. For 10H surfaces, we found a less negative surface charge compared to FLAT, due to the effect of the linear pattern. The effects of peaks and valleys onto zeta-potential measurements have been already described [63]. The fact that linear patterns retain charges inside the valleys prevents their detection at the end of the channel and possibly generates an opposite flow, leading to a decrease in the measured zeta potential. 

### 3.2. Biofunctionalization of CoCr and Surface Characterization

The studied peptides were synthesized with the structure and the characteristics presented in Figure 1 and Table 1. The peptides contain, first, the bioactive sequences RGD and YIGSR (in blue in Figure 1), which determine the biological functionality of the biomolecule. The RGD sequence was chosen for its well-known effect on cell adhesion [19,22,24], and YIGSR was selected for its ability to bind specifically to ECs [26,29,31,32,33]. Secondly, the spacer units, three aminohexanoic acids (Ahx) (in red), ensure the appropriate accessibility of the functional sequences to interact with cells [32,64]. Finally, two lysine amino acids (in green) contain primary amines in their side chains as anchoring groups to covalently attach the peptides to the silanized surface.

**Figure 3:**
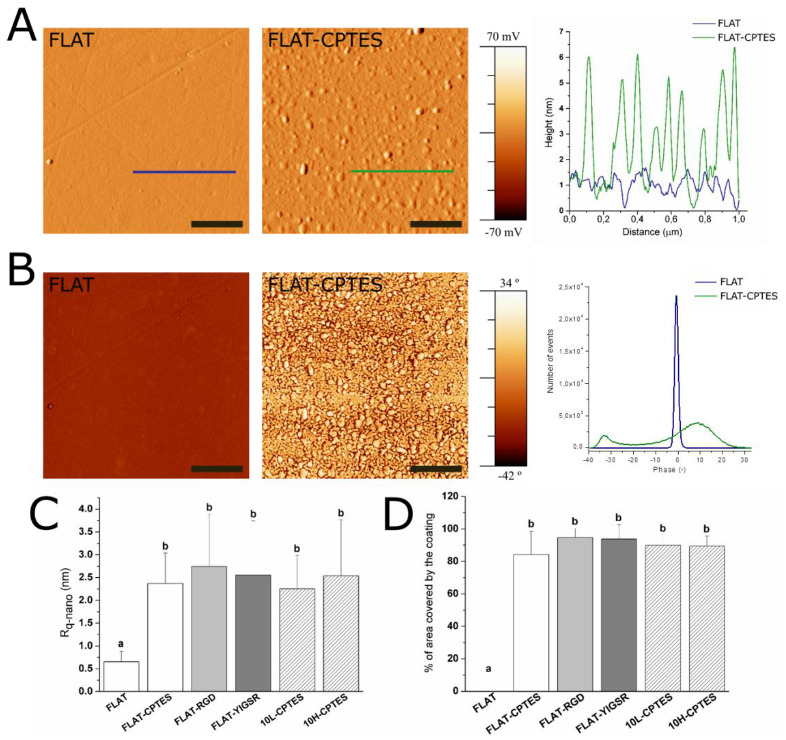
AFM analysis of FLAT, FLAT–CPTES, FLAT–RGD, FLAT–YIGSR, 10L–CPTES and 10H–CPTES surfaces to characterize the silane and peptides layers. Scale bar = 500 nm. Columns marked with different letters (a, b) belong to statistically different groups (*p*-value < 0.05). (**A**) AFM error signal mode images and topographical profiles of FLAT and CPTES surfaces. (**B**) Tapping-mode AFM phase images and phases distributions of FLAT and CPTES surfaces. (**C**) Roughness Rq-nano calculated from AFM height images. (**D**) Percentage of area covered by the coating calculated from AFM phase images.

**Figure 4:**
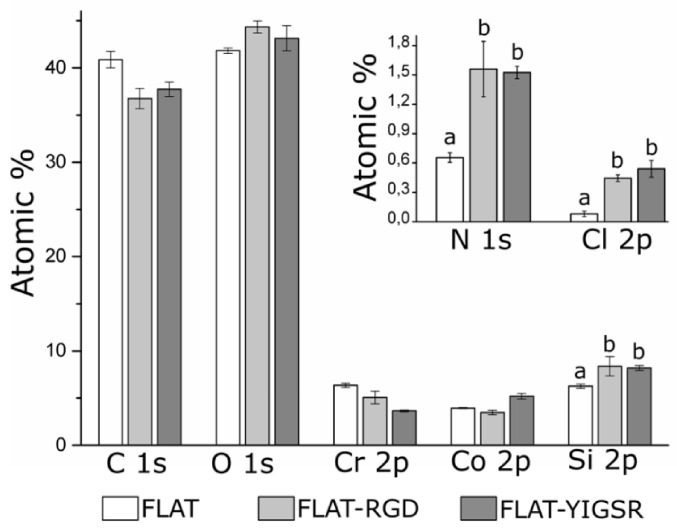
Analysis of the chemical composition (atomic%) of FLAT, FLAT–RGD and FLAT–YIGSR surfaces by XPS. For Si 2p, N 1s and Cl 2p, bars designated with different letters (a, b) belong to statistically different groups (*p*-value < 0.05).

The effectiveness of silanization and functionalization of CoCr surfaces was evaluated by ATR–FTIR (Supplementary Figure S1), AFM (Figure 3) and XPS (Figure 4). ATR–FTIR spectra of FLAT–CPTES, FLAT–RGD and FLAT–YIGSR surfaces are presented in Supplementary Figure S1. On all the surfaces, the peak observed at 1267 cm^−1^, which corresponds to C=O bonding, was the result of atmospheric contamination. On CPTES samples, Si-O-R peaks, at 1100 cm^−1^, were related to the covalent bonding of the silane on the surface [65,66]. Moreover, the terminal chlorine group from the CPTES silane was detected at a wavelength of 870 cm^−1^. Finally, as CoCr alloys did not contain CH_3_ group, the CH_3_ peak, at 1430 cm^−1^, confirmed the presence of CPTES on FLAT–CPTES surfaces. The spectra of RGD- and YIGSR-coated surfaces displayed exactly the same peaks detected on CPTES samples. In particular, no N bonding peaks were detected, as it would be expected in the presence of peptides. This observation could be due to the low sensitivity of the surface technique, which would be not adequate to detect a low amount of biomolecule. 

AFM characterization was performed to analyze the layer of silane and peptides immobilized onto CoCr surfaces (Figure 3). As expected, FLAT surface showed a smooth surface with some scratches from polishing, while CPTES series presented small clusters of approximately 6 nm in height (Figure 3A). Similar differences were observed by Sargeant et al. [67] after silanization on NiTi alloy surfaces. Moreover, FLAT–RGD, FLAT–YIGSR, 10L–CPTES and 10H–CPTES surfaces presented roughness values that were very similar to those measured in the CPTES samples (Figure 3C). Indeed, all five conditions had Rq-nano values of about 2.5 nm with no statistical differences, whereas FLAT samples presented values that were four times lower (Rq-nano = 0.66 ± 0.23 nm). The increment of the Rq-nano roughness for silanized and functionalized surfaces, compared to FLAT, can thus be attributed to the coating layer. Since no statistical differences were found between silanized and functionalized surfaces, we can assume that the peptide layer is very thin and not observable with AFM. Moreover, the similar roughness observed on FLAT and patterned silanized surfaces suggested that the efficiency of silanization is not affected by the DLIP process. 

Figure 4B exhibits the phase map for the region of interest. It shows a single phase for FLAT surfaces, while CPTES series showed a bimodal phases distribution at −33° and 8°. AFM phase imaging in tapping mode is directly related to the viscoelastic properties of the analyzed surface and, consequently, to the surface hardness of the material. Thus, CPTES surfaces seemed to have different materials properties that are presumably related to the immobilization of silanes onto CoCr surface. Furthermore, the percentage of covered area by the coating was calculated (Figure 3D). All functionalized surfaces presented 85% surface coverage, and no statistical differences were observed between silanized and functionalized surfaces for both polished and patterned CoCr surfaces.

To evaluate the presence of immobilized peptides onto functionalized surfaces, FLAT, FLAT–RGD and FLAT–YIGSR surfaces were characterized by XPS (Figure 4). Untreated CoCr alloys displayed characteristic peaks of C 1s, O 1s, Co 2p and Cr 2p. The presence of carbon is attributed to atmospheric organic contaminants [68]. A small amount of nitrogen (N 1s) and silicon (Si 2p) contaminations was also detected. On FLAT–RGD and FLAT–YIGSR surfaces, the percentage of Si 2p was increased due to the presence of CPTES. A significant increase in N 1s was also detected for functionalized surfaces compared to FLAT (FLAT, 0.66 ± 0.05%; FLAT–RGD, 1.56 ± 0.28%; FLAT–YIGSR, 1.53 ± 0.03%). This presence of nitrogen was attributed to the amide groups and other amino functionalities characteristic of the peptide sequences [69], thereby confirming the attachment of the RGD and YIGSR peptides to the surfaces. However, the fact that Cl 2p was detected, as well, after the functionalization indicates that not all the electrophilic points of CPTES (i.e., –CH_2_-Cl) reacted with the peptides. This observation suggests a limited coverage of the silane layer by the peptides, in agreement with the low% of N 1s quantified and FTIR data. Finally, it is worth mentioning that no statistical differences were observed in N 1s, Si 2p and Cl 2p signals between FLAT–RGD and FLAT–YIGSR surfaces, suggesting that the functionalization occurred with the same effectiveness and that the amount of immobilized peptides was very similar. 

**Figure 5:**
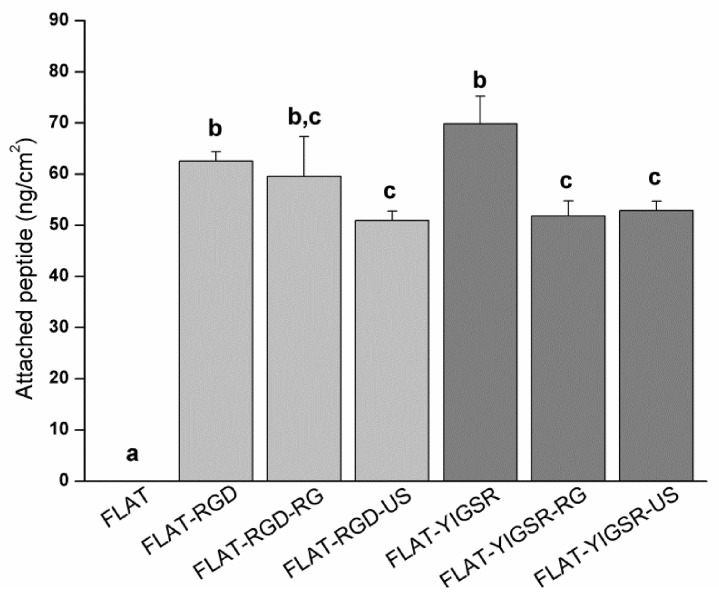
Quantification by indirect fluorescence of the amount of immobilized peptides onto FLAT and biofunctionalized surfaces before (FLAT–RGD and FLAT–YIGSR) and after sterilization with gamma radiation (FLAT–RGD–RG and FLAT–YIGSR–RG) or after ultrasound sonication (FLAT–RGD–US and FLAT–YIGSR–US). Bars designated with different letters (a, b, c) belong to statistically different groups (*p*-value < 0.05).

To evaluate the stability of the covalent immobilization of the different peptides, FLAT–RGD and FLAT–YIGSR surfaces were subjected to either gamma radiation sterilization or ultrasound sonication with Milli-Q water, and the amount of immobilized fluorescent peptide was quantified before and after the stability treatments (Figure 5). The amount of immobilized fluorescent peptide onto FLAT–RGD and FLAT–YIGSR was calculated before the stability treatments and was statistically the same, confirming the results observed with XPS. The amount of immobilized peptide slightly decreased for both peptides after the stability tests, although the percentages of peptide loss were very moderate and, overall, 74% or more of the peptides remained on the surface (FLAT–RGD–RG, 95.2%; FLAT–RGD–US, 81.5%; FLAT–YIGSR–RG, 74.2%; and FLAT–YIGSR–US, 75.7%). Ultrasonication represents an aggressive mechanical challenge and may remove physisorbed peptides attached through weak bonds. Gamma radiation could affect chemical covalent or weak bonding and, consequently, remove a piece or the entire peptide [70]. It should be noted that the CF fluorescent molecule was coupled at the N-terminus of the peptide sequence, i.e., at the opposite of the anchoring regions of the C-terminus; thus, the detection of fluorescence after the RG sterilization indicates that more than 74% of attached peptides remained entire. These results suggest that a majority of the peptide coatings are stable to RG sterilization and mechanical stress, which both occur during stent implantation.

### 3.3. HUVECs Adhesion and Alignment

**Figure 6:**
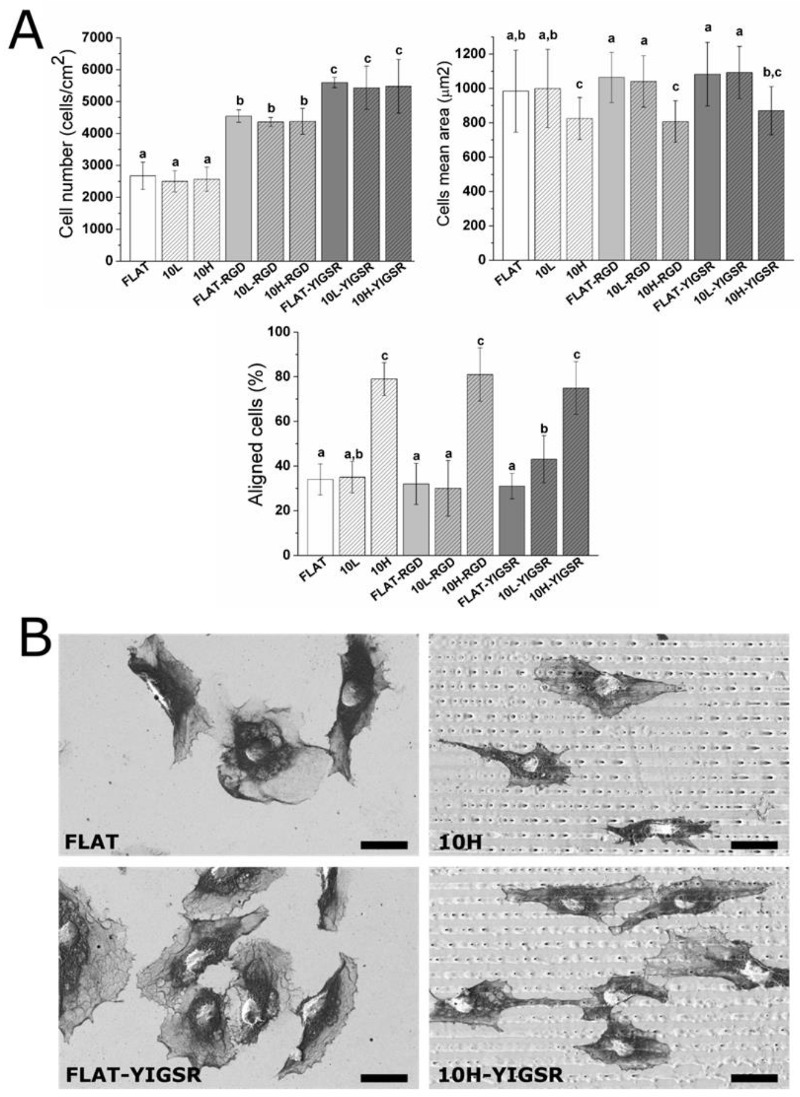
HUVECs adhesion and spreading on FLAT, patterned or/and functionalized CoCr surfaces after 12 h of incubation. (**A**) Cell number, cells mean area and % of aligned cells were obtained by fluorescent microscopy. Images were analyzed with FIJI software. Bars designated with different letters (a, b, c) belong to statistically different groups (*p*-value < 0.05). (**B**) FE-SEM images of HUVECs on FLAT, 10H, FLAT–YIGSR and 10H–YIGSR surfaces. Scale bar = 30 μm.

The number of adhered HUVECs onto modified CoCr surfaces was evaluated by using fluorescent microscopy (Figure 6A). Polished and patterned non-functionalized surfaces presented the same number of adherent cells. Previous studies have reported higher cell adhesion on linear-patterned surfaces with the same periodicity (i.e., around 10 µm) [35,40,71,72,73]. However, the investigations on DLIP-patterned surfaces described no changes in cell adhesion [33,74], possibly as a result of the relatively low pattern depth (<1 µm) introduced by this technique and the non-sharp shape of the patterns peaks. Differences in cell area were not observed between FLAT and 10L surfaces. In contrast, this parameter was statistically reduced with 10H. The reason for such behavior could probably be attributed to the effect of the peaks geometry that acted as a barrier for cellular spreading. Thus, we considered that high-depth DLIP patterning had no effect on cell number but slightly reduced the spreading of attached HUVECs. 

On the contrary, surface functionalization with the peptides did not modify the cell mean area but significantly increased the number of attached HUVECs, with YIGSR functionalization yielding the highest values of cell adhesion. Indeed, for RGD and YIGSR functionalized surfaces, cell numbers increased by approximately 80% and 120%, respectively, compared to the non-functionalized surfaces. The observed increased number of adhered cells for YIGSR, combined with the fact that this peptide sequence specifically targets ECs [25,27,29], indicates that CoCr functionalization with YIGSR peptides is a feasible way to promote ECs adhesion, while avoiding the adhesion of SMCs or platelets [32].

Next, HUVECs’ alignment was characterized by fluorescent staining of actin fibers (Figure 6A) and FE-SEM visualization (Figure 6B). A cell was considered aligned if the angle between the patterned lines and the longest segment of the cell was inferior to 30°. Consequently, a surface having no effect on the ECs’ orientation should present around 33% of aligned cells. HUVECs’ alignment was directly related to the pattern dimension. FLAT series and 10L-patterned series, functionalized or not, produced a percentage of HUVECs’ alignment between 31 and 40%, and thus had little impact on cellular alignment. The designed patterning of 10H surfaces, however, induced a remarkable alignment of HUVECs of 79 ± 7%, 81 ± 12% and 75 ± 12% for 10H, 10H–RGD and 10H–YIGSR, respectively, without statistical differences within them. Thus, the 10H series induced the alignment of HUVECs following the direction of the patterns and reduced HUVECs’ mean area, due to the micro-level of the pattern. Patterns of 10L surfaces had grooves with a depth that was 10 times smaller than the ECs’ thickness [75], and it is probable that they simply act as a pavement instead of a wall where the HUVECs can easily spread in all directions.

### 3.4. HUVECs Migration

**Figure 7:**
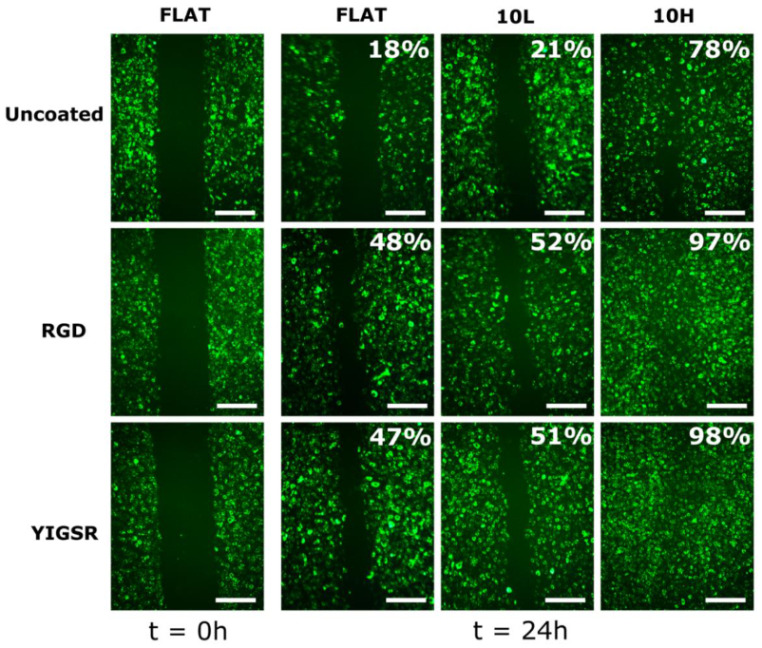
HUVECs’ migration on FLAT, patterned and/or functionalized CoCr surfaces at 0 and 24 h time points. The percentage indicates the percentage of the respective initial injury area covered by HUVECs at 24 h. Initial gap width = 500 ± 50 µm. Scale bar = 400 µm.

Cell migration was evaluated through the injury coverage ratio, which was defined as the percentage of injury area covered by HUVECs after 24 h (Figure 7). The migration of HUVECs was observed on all CoCr surfaces. On non-functionalized FLAT and 10L surfaces series, the injury recovery was only 18% and 21%, respectively, demonstrating that 10L patterns did not have an effect on HUVECs migration. On the contrary, 10H patterns presented a higher value of 78% of recovery, indicating an accelerated migration, probably due the alignment produced by the patterned topography [35,71,76,77]. When FLAT and 10L CoCr surfaces were functionalized with RGD and YIGSR peptides, HUVECs migration was significantly accelerated (more than 2-fold), with no differences detected between the two peptides. It is noteworthy that the combination of 10H patterns and functionalization with RGD and YIGSR peptides significantly improved HUVECs’ migration compared to all surfaces: 10H RGD and 10H YIGSR surfaces presented an almost complete healing of 97% and 98%, respectively. This result proves that combining DLIP patterning and functionalization with RGD or YIGSR peptides, which increases HUVECs’ alignment and adhesion, respectively, synergistically accelerates HUVECs’ migration after 24 h.

### 3.5. HUVECs Proliferation

**Figure 8:**
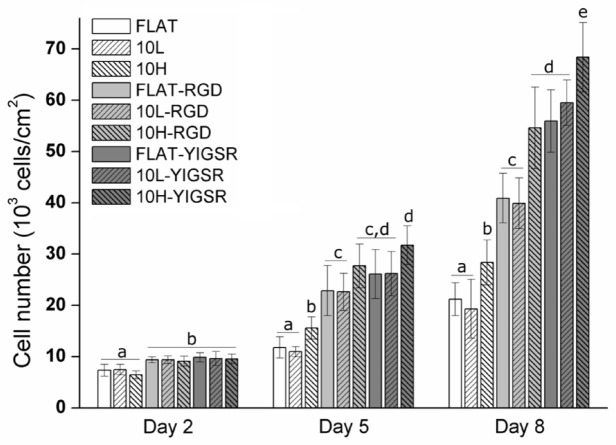
HUVECs’ proliferation on FLAT, patterned and/or functionalized CoCr surfaces at 2, 5 and 8 days of cell culture obtained by Alamar blue assay. Bars designated with different letters (a, b, c, d, e) belong to statistically different groups (*p*-value < 0.05).

The effect of DLIP and RGD and YIGSR functionalization on HUVECs proliferation was assessed by an Alamar blue assay after 2, 5 and 8 days of cell culture (Figure 8). As expected, HUVECs’ proliferation increased on all surfaces over time. After 2 days, the cell number was significantly higher on all peptide functionalized surfaces compared to non-functionalized FLAT, 10L and 10H surfaces. After 5 and 8 days, this effect was further emphasized. Moreover, linear patterning had a significant effect, and, at 5 and 8 days, a higher cell proliferation was observed for the 10H series compared to the respective FLAT and 10L surfaces. This was probably induced by the faster cell migration observed on these patterns. Indeed, when proliferating, cells have less space and are in close contact with other cells. Consequently, due the effect of contact inhibition of proliferation [78,79], proliferation slows down. On a surface generating a faster migration, such as 10H surfaces, cells tend to separate from the others faster and, consequently, proliferation is accelerated compared to surfaces with a slower migration rate, such as FLAT and 10L surfaces. Finally, after 8 days, surfaces functionalized with YIGSR showed a higher cell number compared to the respective surfaces functionalized with RGD, corroborating the higher potential of YIGSR to direct HUVEC behavior. 

Each year, millions of patients are treated worldwide with percutaneous coronary intervention, with implantation of a DES or BMS. The use of newer-generation DES, coated with degradable polymers, has been shown to be more effective in the prevention of restenosis than BMS and may also reduce the rate of stent thrombosis [56]. However, the evidence in favor of newer-generation DES over BMS may not be as strong as it was originally thought to be, mainly due to the associated long-term risk of DES compared to BMS [1,2]. In fact, it has been identified a higher risk of stent thrombosis in patients receiving first-generation DES, coated with inert polymers, than in those receiving BMS [80,81]. The main reason is the low capability of the implants to re-endothelialize the surface in a short period of time, i.e., between 6 and 9 months. The present work opens an opportunity to translate the obtained results to commercial BMS to specifically overcome the low rate of re-endothelialization with a combined surface treatment designed to ameliorate ECs’ migration and proliferation and, thus, possibly minimize long-term risks. Overall, cell studies determined that peptide functionalization increased ECs’ adhesion and 10H DLIP patterning induced HUVECs alignment. It is noteworthy that the synergy of these two effects on 10H–RGD and 10H–YIGSR surfaces accelerated HUVECs’ migration and proliferation. Blood circulating in the artery contains a low quantity of ECs. Consequently, the adhesion of ECs on the stent, which would be required to accelerate arterial regeneration, is limited, and, therefore, ECs migration from the surrounding healthy tissue plays a crucial role in the re-endothelialization process [82,83]. In addition, previous studies have demonstrated that DLIP-patterned CoCr surfaces are anti-thrombogenic [33] and that CoCr alloys functionalized with YIGSR do not promote high levels of SMC adhesion [32]. Therefore, CoCr alloys patterned by DLIP and functionalized with YIGSR seem to have a great potential to accelerate endothelium healing without generating platelet aggregation and SMC proliferation.

## 4. Conclusions

In this work, we specifically explored the possibility of increasing ECs’ adhesion, migration and proliferation on CoCr surfaces by combining topographical modification and biofunctionalization techniques.

CoCr surfaces were successfully modified by using the DLIP technique, obtaining linear patterned surfaces. These topographies did not modify the corrosion properties and the surface charge of the CoCr alloy.

To evaluate the combined effect of topographical modifications with biofunctionalization, RGD and YIGSR cell adhesive peptides were covalently immobilized to the surfaces through CPTES silanization. Cell studies indicated that RGD- and YIGSR-coated surfaces induce a significant increase in ECs’ adhesion compared to non-functionalized surfaces. Moreover, 10H pattern surfaces aligned the 80% of adhered ECs, while 10L did not affect cell alignment. When 10H patterned surfaces were functionalized, an accelerated ECs migration and proliferation was observed. Consequently, a positive synergy between surface linear patterning and functionalization was described. Therefore, DLIP patterned surfaces functionalized with RGD or YIGSR hold great potential to overcome clinical limitations of current stents by enhancing surface endothelialization.

## Figures and Tables

**Table 1:** RGD, RGD–CF, YIGSR and YIGSR–CF peptides sequences, retention time (tR), purity and molecular masse (*m*/*z*) obtained by analytical HPLC and MALDI–TOF.

Code	Sequence	*t*_R_ (min) ^a^	Purity (%) ^a^	Calculated *m*/*z*	Experimental *m*/*z* ^d^ [M + H]^+^
**RGD**	Ac-Arg-Gly-Asp-Ser-(Ahx)_3_-Lys-Lys-NH_2_	2.955 ^b^	93	1069.66	1069.68
**RGD–CF**	CF-Arg-Gly-Asp-Ser-(Ahx)_3_-Lys-Lys-NH_2_	4.345 ^b^	98	1385.70	1385.66
**YIGSR**	Ac-Tyr-Ile-Gly-Ser-Arg-(Ahx)_3_-Lys-Lys-NH_2_	5.228 ^c^	98	1230.78	1230.75
**YIGSR–CF**	CF-Tyr-Ile-Gly-Ser-Arg-(Ahx)_3_-Lys-Lys-NH_2_	5.503 ^c^	99	1546.82	1546.80

^a^ Retention time (tR) and purity were calculated by analytical HPLC, using a reversed-phase XBridge BEH130 C-18 column (4.6 mm × 100 mm, 3.5 µm) (Waters, Milford, MA, USA) and a photodiode array detector (Waters 2998). ^b^ Linear gradient: 0 to 60% MeCN over 8 min; flow, 1.0 mL/min. ^c^ Linear gradient, 5 to 100% MeCN over 8 min; flow, 1.0 mL/min. ^d^ Mass spectra were recorded on a MALDI–TOF Voyager DE RP spectrometer (Applied Biosystems, Foster City, CA, USA).

**Table 2:** Topographical parameters of the FLAT, 10L and 10H surfaces. Rq-micro is the root-mean-square roughness measured on a 50 × 50 µm^2^ area. Rq-nano is the root-mean-square roughness measured on a 1 × 1 µm^2^ area.

	Periodicity (P)	Depth (D)	Rq-micro		Rq-nano
	µm	nm	nm		nm
**FLAT**	-	-	7 ± 1	-	0.66 ± 0.23
				Valley	0.54 ± 0.17
**10L**	10.0 ± 0.1	79 ± 12	33 ± 4	Peak	0.78 ± 0.31
				Valley	0.79 ± 0.22
**10H**	10.0 ± 0.1	762 ± 83	304 ± 25	Peak	0.79 ± 0.17

**Table 3:** Detailed results of corrosion resistance, ion release and zeta potential on CoCr surfaces before and after DLIP patterning. Corrosion potential and ion release were obtained according to the standard ISO 10993-15. Isoelectric point (IEP) and zeta potential (ζ) at pH 7.4 were measured.

	Corrosion Potential	Ion Release			Zeta Potential	
		Co Ions	Cr Ions	Ni Ions	IEP	ζ at pH 7.4
	mV	ng	Ng	ng	pH	mV
**FLAT**	1014.3 ± 10.1	47.5 ± 16.0	1.6 ± 0.3	12.4 ± 3.8	4.9 ± 0.0	−34.5 ± 2.9
**10L**	1017.6 ± 9.5	23.3 ± 6.3	1.5 ± 0.2	9.4 ± 2.8	4.8 ± 0.0	−39.7 ± 0.2
**10H**	1031.7 ± 6.1	77.8 ± 8.6	4.3 ± 0.3	17.5 ± 1.0	4.5 ± 0.0	−11.5 ± 1.0

## Data Availability

Th data presented in this study are available on request from the corresponding author.

## Supplementary Materials

The following are available online at https://www.mdpi.com/article/10.3390/nano12071217/s1. Figure S1: ATR–FTIR of FLAT–CPTES, FLAT–RGD and FLAT–YIGSR surfaces from 700 to 1600 cm^−1^. Arrows indicate significant peaks.
