# Supplementary material for: Effectiveness of Direct Laser Interference Patterning and Peptide Immobilization on Endothelial Cell Migration for Cardio-Vascular Applications: An In Vitro Study
Source: Nanomaterials (Basel). 2022 Apr 5;12(7):1217. doi: 10.3390/nano12071217 (PMC9002369; doi:10.3390/nano12071217)
Supplement: Supplementary file 1 [file nanomaterials-12-01217-s001.zip › nanomaterials-1549460-supplementary.pdf]

nanomaterials

Supplementary Material for:

Effectiveness of Direct Laser Interference Patterning and Peptide Immobilization on Endothelial Cell Migration for Cardio-Vascular Applications: An In Vitro Study

Romain Schieber [1,2,3], Carlos Mas-Moruno [1,2], Federico Lasserre [3], Joan Josep Roa [2,4], Maria-Pau Ginebra [1,2,5], Frank Mücklich [3] and Marta Pegueroles [1,2,*]

1 Biomaterials, Biomechanics and Tissue Engineering Group, Department of Materials Science and Engineering, Universitat Politècnica de Catalunya (UPC), EEBE, Av. Eduard Maristany, 10-14, 08019 Barcelona, Spain; romain.schieber@upc.edu (R.S.); carles.mas.moruno@upc.edu (C.M.-M.); maria.pau.ginebra@upc.edu (M.-P.G.)
2 Barcelona Research Center in Multiscale Science and Engineering, Universitat Politècnica de Catalunya (UPC), 08019 Barcelona, Spain; joan.josep.roa@upc.edu
3 Chair of Functional Materials, Faculty of Natural Sciences and Technology, Saarland University, 66123 Saarbrücken, Germany; lasserre@matsci.uni-sb.de (F.L.); muecke@matsci.uni-sb.de (F.M.)
4 Structural Integrity, Micromechanics and Reliability of Materials Group, Department of Materials Science and Metallurgical Engineering, UPC, EEBE, 08019 Barcelona, Spain
5 Institute for Bioengineering of Catalonia (IBEC), 08028 Barcelona, Spain
* Correspondence: marta.pegueroles@upc.edu (M.P.); Tel.: +34-934054154

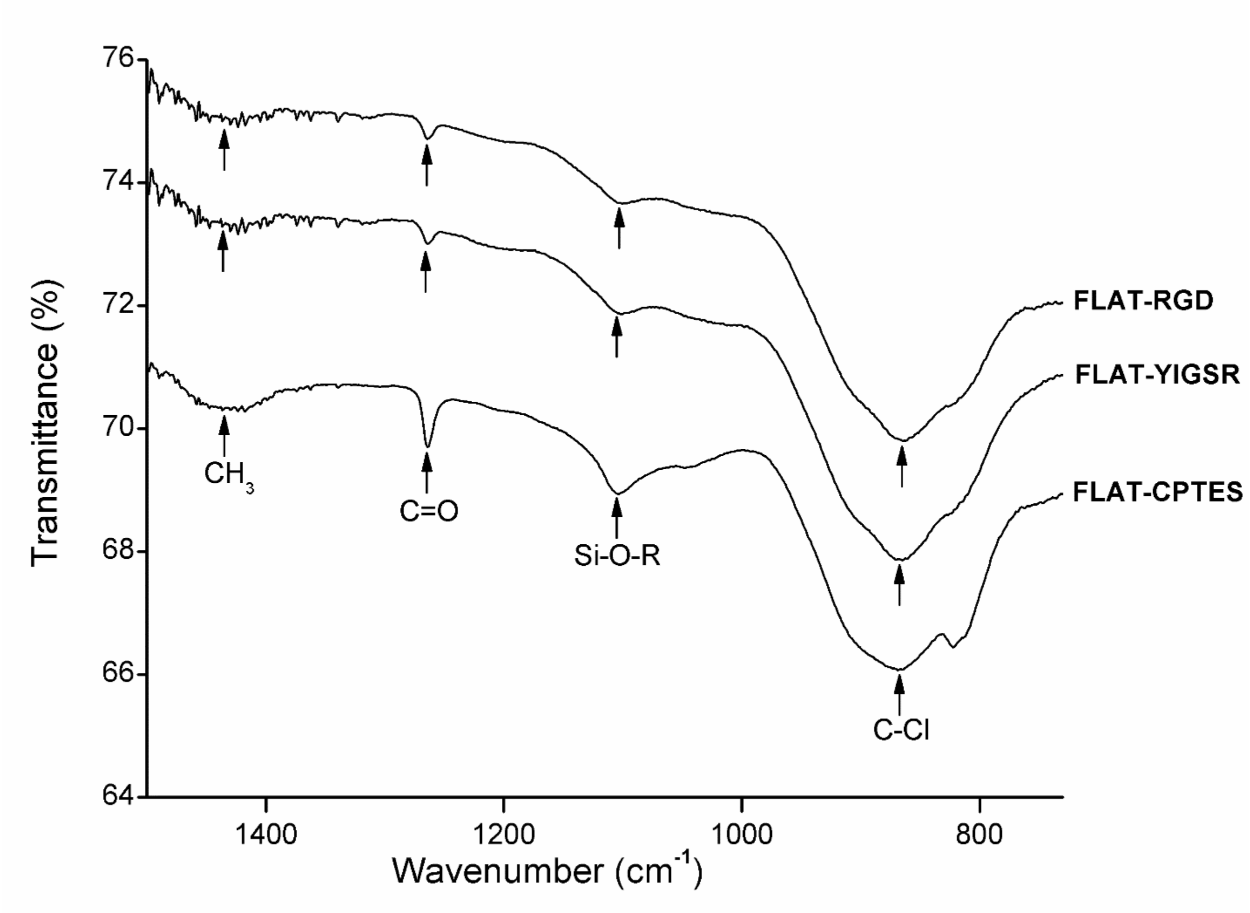

Figure S1. ATR-FTIR of FLAT-CPTES, FLAT-RGD and FLAT-YIGSR surfaces from 700 to 1600 cm^{-1}. Arrows indicate significant peaks.
